# Synergistic effects of HO-1 inhibition and chemotherapy on tumor proliferation and immune infiltration: An *in vitro* and *in vivo* approach to enhancing prostate cancer treatment

**DOI:** 10.1016/j.tranon.2025.102339

**Published:** 2025-03-03

**Authors:** Ramia J. Salloom, Dania Z. Sahtout, Iman M. Ahmad, Maher Y. Abdalla

**Affiliations:** a Department of Pathology, Microbiology, and Immunology, USA; b Department of Clinical, Diagnostics, and Therapeutic Sciences, University of Nebraska Medical Center, Omaha, NE, USA

**Keywords:** Heme oxygenase-1 (HO-1), Prostate cancer (PC), ZnPP, SnPP, and Docetaxel (Doc)

## Abstract

Prostate cancer (PC) remains a leading cause of morbidity and mortality among men worldwide, highlighting the need for novel therapeutic strategies. Our study investigates the therapeutic potential of targeting the heme degradation pathway through heme oxygenase-1 (HO-1) inhibition in PC. Using both *in vitro* and *in vivo* models, we explored the effects of combining HO-1 inhibition with chemotherapy, represented by docetaxel (Doc), on tumor growth and immune infiltration. *In vitro* experiments demonstrated that HO-1 inhibition, as well as HO-1 knockout (KO), significantly reduced tumor cell proliferation and enhanced chemosensitivity in RM-1 cells. Additionally, U937 cells co-cultured with HO-1 KO cells shifted cell polarization toward an M1 phenotype. *In vivo*, the combined treatment of the HO-1 inhibitor, tin protoporphyrin (SnPP), with Doc significantly enhanced anti-tumor efficacy in mouse models compared to chemotherapy or SnPP alone. This combination therapy not only reduced Ki67 expression and increased CC3 expression in tumor tissues but also shifted macrophage polarization toward an M1 phenotype and enhanced CD4^+^ and CD8^+^ T cells infiltration, indicating an augmented immune response. Further investigation using macrophage-specific HO-1 knockout mice revealed a direct role of HO-1 inhibition in driving macrophage polarization, confirming its involvement in promoting the shift toward an M1 phenotype. Although this response was significant, it was more robust with systemic HO-1 inhibition. Our findings indicate that HO-1 inhibition can potentiate the effects of chemotherapy, offering a promising avenue for improving PC treatment outcomes.

## Highlights

• HO-1 inhibition decreases prostate cancer cell viability and enhances sensitivity to docetaxel both *in vitro* and *in vivo*.
• Combining HO-1 inhibition with docetaxel significantly reduces Ki67 expression, indicating decreased tumor proliferation.
• The combination therapy markedly increases cleaved caspase-3 (CC3) expression, promoting apoptosis in tumor tissues.
• HO-1 inhibition combined with docetaxel boosts CD4^+^ and CD8^+^ T cells infiltration, enhancing the immune response within tumor tissues.
• This therapeutic combination also shifts the tumor microenvironment towards an M1 macrophage phenotype, supporting anti-tumor immunity**.**

## Graphical abstract

**Figure:**
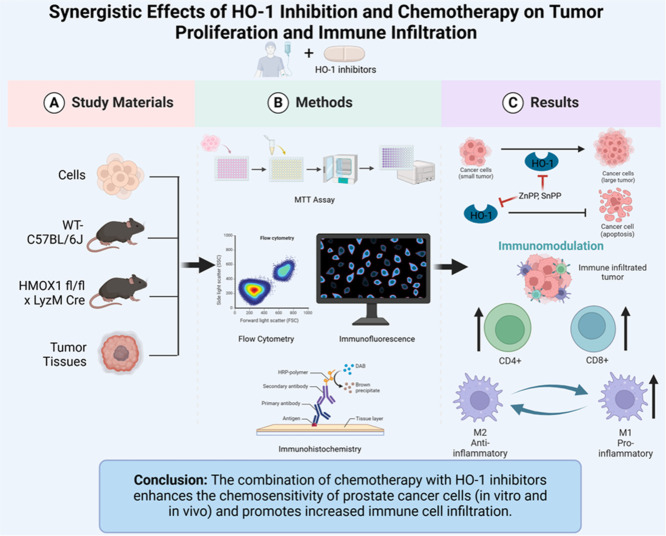
Synergistic effects of HO-1 inhibition and chemotherapy on tumor proliferation and immune infiltration. A) Study materials included *in vitro* prostate cancer cells, WT C57BL/6J mouse models subcutaneously implanted with RM-1 parent or RM-1 HO-1 KO cells, HMOX1 fl/fl xLyzM Cre mouse model subcutaneously implanted with RM-1 parent cells, and tumor tissues collected from various treatment groups across the different mouse models. B) Methods utilized in the study were: MTT assay to assess cellular viability and chemosensitivity *in vitro*, Flow cytometry to analyze M1 and M2 macrophage polarization *in vitro*, Immunofluorescence microscopy to evaluate the effect on macrophage polarization in tumor tissues, and Immunohistochemistry to determine protein expression and immune infiltration in tumor tissues. C) Results demonstrated that HO-1 inhibition reduces PC cell viability, enhances apoptosis, and promotes ant-tumor immune infiltration, characterized by increased CD4^+^ and CD8^+^ T cells infiltration and a shift toward M1 macrophage polarization. (Created with Biorender.com).

## Introduction

PC is one of the most common malignancies among men, representing a significant global health burden [1,2]. Despite advances in early detection and treatment, the disease often progresses to an advanced, treatment-resistant stage, posing substantial challenges for effective management [3,4]. Current therapeutic strategies for advanced PC primarily involve androgen deprivation therapy (ADT), chemotherapy, and targeted therapies [[5], [6], [7], [8]]. However, these treatments frequently encounter resistance, necessitating the development of novel approaches to improve patient outcomes.

Recent advancements in systemic treatments for PC have highlighted the importance of incorporating predictive biomarkers and personalized therapeutic strategies to optimize patient outcomes [[9], [10], [11]]. Biomarkers such as AR-V7 expression and homologous recombination repair (HRR) gene mutations have emerged as predictors of response to androgen receptor-targeted therapies and poly (ADP-ribose) polymerase (PARP) inhibitors, respectively [12,13]. Moreover, studies have explored the interplay between the tumor microenvironment, immune modulation, and systemic therapies, shedding light on potential combination strategies to overcome resistance [[14], [15], [16], [17]]. These evolving concepts underline the need for therapies that address both intrinsic tumor properties and the dynamic tumor-immune interactions to achieve durable responses.

Doc is a chemotherapy agent commonly used to treat advanced PC, especially in cases where the disease has become resistant to initial treatments [[18], [19], [20]]. It works by stabilizing microtubules, thereby inhibiting cell division, and inducing apoptosis in cancer cells [18,21]. While Doc has improved survival rates for many patients, its effectiveness is often limited by the development of resistance and its significant side effects [19,20,22]. This underscores the need for combination therapies that can enhance its efficacy and reduce adverse reactions.

HO-1 has emerged as a potential therapeutic target in cancer due to its multifaceted roles in promoting tumor growth, metastasis, and resistance to therapy [23,24]. HO-1 is an enzyme that catalyzes the degradation of heme into biliverdin, free iron (Fe^2+^), and carbon monoxide (CO) [23,25]. While this enzymatic activity plays a critical role in cellular defense against oxidative stress, its overexpression in various cancers, including PC, has been linked to enhanced tumorigenicity and poor prognosis [8,23,26]. In addition to its enzymatic functions, HO-1 has non-canonical roles in cancer progression [27,28]. Notably, HO-1 can translocate to the nucleus, where it influences gene expression and promotes tumor survival [29]. Nuclear HO-1 has been implicated in regulating cellular proliferation, angiogenesis, and metastasis, contributing to its role in cancer progression and therapy resistance [30,31]. The nuclear translocation of HO-1 adds another layer of complexity to its role in cancer, making it a particularly compelling target for therapeutic intervention.

HO-1 inhibitors have been developed to target the pro-tumorigenic activities of the enzyme, aiming to disrupt its protective effects on cancer cells. By blocking the enzymatic activity of HO-1, these inhibitors prevent the degradation of heme into biliverdin, Fe^2+^, and CO [23,32]. Preclinical studies have shown that several small-molecule inhibitors of HO-1 can reduce tumor growth, enhance the efficacy of chemotherapeutic agents, and induce apoptosis in cancer cells [31,33].

Our lab previously showed that HO-1 inhibition improved the responsiveness of pancreatic cancer cells to chemotherapy [34]. Similarly, in PC, studies from our lab and others have demonstrated that HO-1 inhibition reduces cell proliferation, increases oxidative stress, and enhances chemosensitivity [[35], [36], [37]]. These effects suggest that targeting HO-1 disrupts the antioxidant defenses of cancer cells, rendering them more vulnerable to the cytotoxic effects of conventional therapies like chemotherapy.

HO-1′s inducible nature allows for targeted inhibition using metalloporphyrins (MPs), which are compounds structurally similar to heme but with a metal ion replacing the iron, such as zinc protoporphyrin (ZnPP) and tin protoporphyrin (SnPP) [[38], [39], [40]]. MPs bind to HO-1 with a higher affinity than heme, effectively inhibiting its function. Additionally, non-porphyrin-based inhibitors, including small molecules like OB-24 and RNA interference techniques (siRNA and shRNA), are being explored for therapeutic intervention [24,40,41]. By impairing the antioxidant defense mechanism of cancer cells, HO-1 inhibitors can potentiate the effects of conventional treatments and offer a novel therapeutic avenue for combating advanced PC.

The immune system plays a crucial role in tumor surveillance and elimination, with CD8^+^ and CD4^+^ T cells being central to this process. CD8^+^ T cells, also known as cytotoxic T lymphocytes (CTLs), are particularly important for their ability to directly kill tumor cells [[42], [43], [44]]. These cells recognize and bind to antigens presented by major histocompatibility complex (MHC) class I molecules on the surface of tumor cells, leading to the release of cytotoxic granules to induce apoptosis [45,46]. Their infiltration into the tumor microenvironment is strongly associated with improved prognosis and better therapeutic outcomes [16,47]. CD4^+^ T cells, or helper T cells, support this response by secreting cytokines that enhance the activation, proliferation, and memory formation of CD8^+^ T cells, as well as regulating the broader immune response [[48], [49], [50], [51]]. Their role is crucial in maintaining a sustained and effective anti-tumor immune response. Together, these T cells create a coordinated immune network essential for anti-tumor activity, and their enhanced infiltration and function can improve therapy outcomes [47,52].

HO-1 has been shown to modulate the tumor immune landscape, often promoting an immunosuppressive environment that facilitates tumor evasion from immune surveillance [8,16,31]. We hypothesize that Inhibiting HO-1 can reverse these effects, enhancing the infiltration and activity of CD4^+^ and CD8^+^ T cells within the tumor, thereby supporting a more robust and sustained immune response against cancer.

Tumor-associated macrophages (TAMs) are a key component of the tumor microenvironment, being the most abundant inflammatory cell population infiltrating PC tissues [53,54]. PC cells secrete factors such as stem cell factor 1 (SCF-1) and chemokine ligand 2 (CCL2), which recruit monocytes and macrophages to the tumor site [55,56]. Once recruited, these macrophages are exposed to various cytokines in the tumor microenvironment (TME), influencing their polarization into either M1 or M2 phenotypes [55]. M1 macrophages are associated with pro-inflammatory and anti-tumor activities, producing cytokines that support the immune response against cancer cells [57,58]. Conversely, M2 macrophages exhibit anti-inflammatory and pro-tumorigenic functions, promoting tissue remodeling, angiogenesis, and immunosuppression [57,58]. The balance between M1 and M2 macrophage polarization significantly impacts tumor progression and therapy effectiveness [59,60]. HO-1 upregulation has been linked to the M2 phenotype indicating that HO-1 may contribute to macrophage polarization [[61], [62], [63]]. Consequently, targeting HO-1 may shift the balance toward an M1 phenotype, enhancing the anti-tumor immune response and potentially improving therapeutic outcomes.

The present study explores the potential of HO-1 inhibition as a therapeutic strategy in PC. We hypothesize that targeting HO-1 can enhance the efficacy of conventional chemotherapy, thereby improving treatment responses and overcoming resistance. Utilizing both *in vitro* and *in vivo* models, we explored the effects of combining an HO-1 inhibitor with Doc on tumor proliferation, apoptosis, immune infiltration, and macrophage polarization. Our results demonstrate that combining an HO-1 inhibitor with Doc enhances chemosensitivity in PC tumors and increases immune infiltration within the tumor microenvironment, indicating a robust immune response against cancer cells.

Our research provides a comprehensive analysis of the therapeutic potential of HO-1 inhibition in PC, offering insights into a novel combination therapy that could pave the way for improved clinical outcomes in patients with advanced disease. By targeting the heme degradation pathway, we aim to present a viable strategy to enhance the sensitivity of PC to chemotherapy and foster a more effective anti-tumor response.

## Materials and methods

### Cell culture, reagents, and treatments

Mouse prostate carcinoma cells, RM-1 (RRID: CVCL_B459), were cultured in RPMI 1640 medium (Fisher Scientific, Waltham, MA, USA Cat# SH3002702) with 7 % fetal bovine serum (Fisher Scientific, Waltham. MA, USA, Cat# MT35010CV), 1 % penicillin-streptomycin (Fisher Scientific, Waltham, MA, USA, Cat# 15,140,122), and 1 % l-glutamine (Fisher Scientific, Waltham, MA, USA, Cat# 25,030,081). These cultures were maintained in a 37 °C incubator with 5 % CO2. The study utilized several reagents including zinc protoporphyrin (ZnPP) (Santa Cruz Biotechnology, Dallas, TX, USA, Cat# sc-200329A), tin protoporphyrin IX dichloride (SnPP) (Santa Cruz Biotechnology, Dallas, TX, USA, Cat# sc-203452B), Doc (Fisher Scientific, Waltham, MA, USA, Cat# NC9968050), and protoporphyrin IX cobalt chloride (CoPP) (Santa Cruz Biotechnology, Dallas, TX, USA, Cat# sc-294,098). All stock solutions for the *in vitro* experiments were prepared in dimethyl sulfoxide (DMSO).

For our *in vivo* study, SnPP was dissolved in 100 mM NaOH in PBS, with the pH adjusted to 7.4 using 1 N HCl. The solution was then filter-sterilized using a syringe filter, aliquoted, and stored at −80 °C. Doc was prepared fresh for each use through serial dilutions, ensuring that the final DMSO concentration was 5 % of the total volume. It was diluted in a solution containing 5 % DMSO, 30 % PEG300, 5 % Tween 80, and 60 % ddH2O. Each component was added sequentially, making sure the powder was fully dissolved before adding the next reagent.

### HO-1 knockout cells (HO-1 KO)

RM-1 cells (RRID: CVCL_B459) were transduced with lentivirus from VectorBuilder, Inc., Chicago, IL, USA, at a multiplicity of infection (MOI) of 7, following the provided protocol and using 1 μg/mL polybrene to enhance the transduction. Transduced PC cells were then selected in RPMI medium containing 4.0 μg/mL puromycin dichloride in sterile water (Fisher Scientific, Waltham, MA, USA, Cat# AC227420100). Successful transfection was verified through western blot analysis by comparing the effects of CoPP, a pharmacological HO-1 inducer, on the knockout cells *versus* the parental cell lines.

### Western immunoblotting

Protein lysates were prepared using freshly made lysis buffer consisting of 98 % RIPA buffer (Fisher Scientific, Waltham, MA, USA, Cat# PI87787), 1 % protease and phosphatase inhibitor (Fisher Scientific, Waltham, MA, USA, Cat# PI78441), and 1 % EDTA. Protein quantification was performed with a DC Protein Assay Kit (Bio-Rad, Hercules, CA, USA, Cat# 5,000,111) following the manufacturer's protocol and measured using a BioTek Synergy plate reader. Protein lysates (30 μg) were electrophoresed on a 12 % Bis-Tris Gel (BioRad, Hercules, CA, USA, Cat# 4,561,044) at 100 V and transferred to Immobilon-P PVDF membranes (Fisher Scientific, Waltham, MA, USA, Cat# IPVH00010). The membranes were blocked with EveryBlot Blocking buffer (BioRad, Hercules, CA, USA, Cat# 12,010,020). Primary antibodies, including HO-1 pAb (Enzo Life Sciences, Farmingdale, NY, USA, Cat# BML-HC3001–0100, RRID: AB_11,177,779), HO-2 mAb (Cell Signaling Technology, MA, USA, Cat# 32,790, RRID:AB_2,799,030), β- Actin (13E5) rabbit mAb (Cell Signaling Technology, MA, USA, Cat# 4970, RRID:AB_2,223,172), and GAPDH (6C5) (Santa Cruz Biotechnology Dallas, TX, USA, Cat# 32,233, RRID: AB_627,679), were diluted 1:1000 in a 1:1 mixture of EveryBlot blocking buffer and 1 % TBST and incubated overnight at 4 °C. Secondary antibodies, goat anti-rabbit IgG polyclonal (Enzo Life Sciences, Farmingdale, NY, USA, Cat# SAB-300 J, RRID:AB_1,505,668) or mouse IgG HRP-linked (Cell Signaling Technology, MA, USA, Cat# 7076S, RRID:AB_330,924), were diluted 1:3000 in a 1:1 solution of EveryBlot blocking buffer and 1 % TBST, and incubated for 1 hour at room temperature with agitation. Blots were developed using Azure Biosystems Radiance Plus (VWR, Radnor, PA, USA, Cat# 10,147–298) and Azure c600. β-Actin and GAPDH were used as housekeeping genes. Densitometry analysis of western blot bands was performed using ImageJ software.

### Non-radioactive cell proliferation assay (MTT assay)

The MTT cell proliferation assay was performed using the MTT assay kit (Promega, Madison, WI, USA, Cat# G4000), following the protocol provided by the manufacturer. Absorbance was recorded at 570 nm after a 48-hour treatment period using a Tecan Spark 6.0 plate reader.

### Animal studies

The animal experiment protocol was approved by the Institutional Animal Care and Use Committee (IACUC) at the University of Nebraska Medical Center (UNMC) Animal Ethics Committee. All procedures were conducted in accordance with the National Institutes of Health (NIH) guidelines for the care and use of laboratory animals. Mouse models used in our experiments included WT C57Bl/6 J mice (Jackson Laboratories, Bar Harbor, ME, USA) and Hmox1 fl/fl x LyzM Cre (Mac HO-1 KO) mice, kindly provided by Dr. Barbara Wegiel from Harvard Medical School. These mice have a myeloid-specific deletion of HO-1, which were generated as previously described in their published protocol [58,59]. Wild-type C57Bl/6 J mice were selected for their immunocompetence, which allows for the study of immune interactions within the tumor microenvironment and provides a robust model for syngeneic tumor implantation using the RM-1 cell line. Six weeks old male mice were injected with 1 × 10^5 RM-1 parent, or RM-1 KO cells in 100 µL of normal saline into their right flank, creating the following groups: WT-RM-1-parent, WT-RM-1-HO-1-KO, and Mac HO-1-KO-RM-1-parent. Once tumors formed, around day 7, the mice were randomly assigned to different treatment groups. Treatments commenced with mice receiving intraperitoneal injections of 5 mg/kg of SnPP three times per week and/or 10 mg/kg of Doc once weekly for approximately 10 days. Following the treatment period, the mice were sacrificed, and the tumors were harvested and embedded in paraffin blocks.

### Immunohistochemistry (IHC)

Slides were deparaffinized in xylene and rehydrated through a graded series of ethanol concentrations, followed by quenching in 3 % hydrogen peroxide (Fisher Scientific, Waltham, MA, USA, Cat# BP2633500). Antigen retrieval was performed using a 10 mM citrate buffer (pH 6.0) for 15 min. The slides were then blocked with 2.5 % normal horse serum (Vector Laboratories, CA, USA, Cat# S-2012). Primary antibodies, diluted in 2.5 % normal horse serum, were incubated overnight at 4 °C in a humidified chamber. The universal secondary antibody (Vector Laboratories, CA, USA, 30,037) was applied for 1 hour at room temperature. Staining was completed using DAB substrate (Vector Laboratories, CA, USA, Cat# SK-4100) and counterstaining with hematoxylin. The primary antibodies used in our experiments along with their dilutions were Ki67 mAb at 1:300 (Cell Signaling Technology, MA, USA, Cat# 12,202, RRID:AB_2,620,142), CC3 mAb at 1:300 (Cell Signaling Technologies, MA, USA, Cat# 9661, RRID:AB_2,341,188), CD4 mAb (Cell Signaling Technology, MA, USA, Cat# 25,229, RRID:AB_2,798,898), and CD8 mAb (Cell Signaling Technology, MA, USA, Cat# 98,941, RRID:AB_2,756,376).

### Flow cytometry for macrophage polarization

The human monocytic cell line U937 (RRID: CVCL_0007) was plated in a 6-well cell culture plate at a density of 1 × 10^5 cells/well in 10 % RPMI medium. Cells were treated with 100 ng/mL phorbol myristate acetate (PMA) for 24 h to induce differentiation into macrophages. To assess whether factors released from wild-type (WT) parent PC cells and HO-1 KO cells influence macrophage differentiation into M1 or M2 phenotypes, the differentiated macrophages were co-cultured with DU145 parent cells and DU145 HO-1 KO cells using cell culture inserts (Grenier Bio-one, NC, USA, Cat# 657,640) at 37 °C for 24 h. At the end of the 24-hour polarization period, macrophages from all samples (non-treated control, macrophages co-cultured with parent cells, and macrophages co-cultured with HO-1 KO cells) were collected using gentle enzymatic detachment with Accutase (Fisher Scientific, Waltham, MA, USA, Cat# A1110501). The cells were washed with cold PBS, resuspended in flow cytometry staining buffer, and aliquots were stained for 30 min at room temperature, protected from light, with fluorescent conjugated antibodies: CD80 PE (Invitrogen, Cat# 12–0801–81, RRID: AB_465,751), CD206 APC (Invitrogen, Cat# 17–2061–82, RRID: AB_2,637,420), F4/80 Alexa Flour 488 (Invitrogen, Cat# 53–4801–82, RRID: AB_469,915). Samples were then processed at the UNMC Flow Cytometry Core Facility.

### Immunofluorescence (IF)

Slides were deparaffinized in xylene, rehydrated through a series of decreasing ethanol concentrations, and washed with PBS. Antigen retrieval was performed using a 10 mM citrate buffer (pH 6.0) for 15 min. The slides were then blocked with 5 % BSA in distilled water. Primary antibodies, CD206 1:200 (Thermo Fisher Scientific, Waltham, MA, USA, Cat # PA5–101,657, RRID:AB_2,851,091), CD86 1:200 (Thermo Fisher Scientific, Waltham, MA, USA, Cat # PA5–114,995, RRID:AB_2,899,631), and F4/80 1:100 (Thermo Fisher Scientific, Waltham, MA, USA, Cat # GTX26640, RRID:AB_2,865,988), were incubated overnight at 4 °C in a humidified chamber. Two secondary antibodies were used at a dilution of 1:500: Donkey Anti-Rat IgG H&L (Alexa Fluor® 647) preadsorbed anti-mouse (Abcam, Waltham, MA, USA, Cat # ab150155, RRID:AB_2,813,835) and Goat anti-Rabbit IgG (*H* + L) Highly Cross-Adsorbed Secondary Antibody, Alexa Fluor™ 488 (Thermo Fisher Scientific, Waltham, MA, USA, Cat # A-11,034, RRID:AB_2,576,217). These were incubated for 1 hour at room temperature. Vectashield DAPI nuclear staining mounting media (Vector Laboratories, CA, USA, Cat# NC9029229) was used to mount the slides, and images were captured using a Zeiss LSM 710 at the UNMC Advanced Microscopy Core Facility. Quantification analysis was performed using FlowJo software.

### Statistical analysis

Data were analyzed using GraphPad Prism (RRID: SCR_002798) for windows (version 10.0). All data are representative of at least three independent experiments and are presented as the mean ± the standard error of the mean. Pairwise comparisons between groups were performed using one-way analysis of variance (ANOVA) adjusted for three multiple comparisons with Tukey's *post-hoc* test (for *n* > 3) or unpaired *t*-test for *n* = 2. Statistical significance was considered for experiments with a *P* value of <0.05.

### Data availability statement

The data presented in this study are available in this article.

## Results

### Chemotherapy induces HO-1 expression in RM-1 cells

**Fig. 1:**
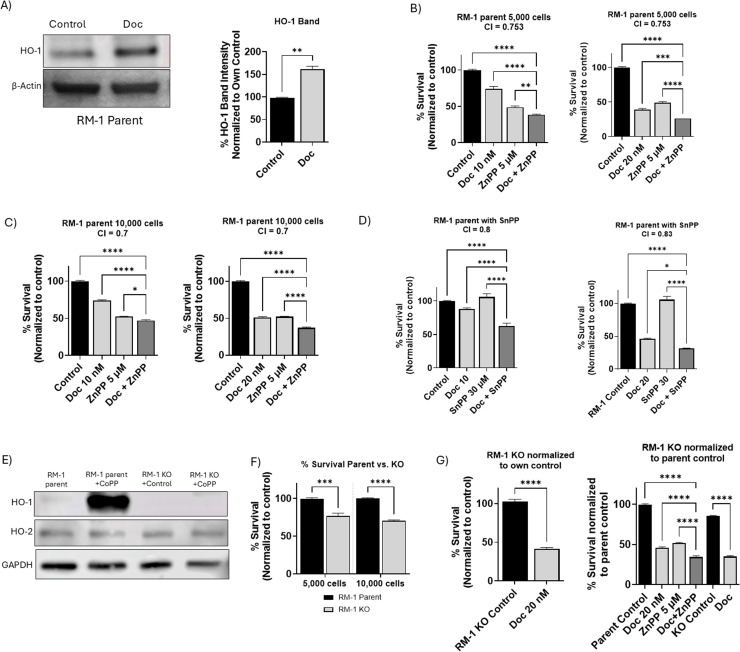
Impact of HO-1 inhibition and HO-1 KO on cellular viability and chemosensitivity in RM-1 cells *in vitro*. (A) Western blot images and densitometric analysis demonstrating HO-1 induction with 20 nM Doc in RM-1 cells. (B) MTT assay results after 48 h showing a significant increase in sensitivity to 10 nM and 20 nM Doc when combined with 5 μM ZnPP in RM-1 cells seeded at a density of 5000 cells/ well. (C) MTT assay results after 48 h showing a significant increase in sensitivity to 10 nM and 20 nM Doc when combined with 5 μM ZnPP in RM-1 cells seeded at a 10,000 cells/ well. (D) MTT assay results after 48 h showing a similar increase in sensitivity with 10 nM and 20 nM Doc combined with 30 μM SnPP. (E) Western blot images confirming HO-1 KO in RM-1 cells employing CoPP (10 μM) as the HO-1 inducer. Consistent HO-2 levels validate the specificity of the KO. (F) MTT assay results after 48 h showing a significant decrease in cellular viability in HO-1 KO RM-1 cells at seeding densities of 5000 and 10,000 cells/ well, compared to parental cells. (G) MTT assay results after 48 h indicating increased sensitivity to 20 nM Doc in HO-1 KO RM-1 cells, normalized to own control and parental control. (*n* = 3, **** = P<0.0001, *** = P<0.0001, ** = P<0.001, * = P<0.05).

Previously, we demonstrated that Doc induces HO-1 expression in human PC cells [64]. To determine whether RM-1 cells, which represent a murine PC cell line, exhibit a similar response, cells were treated with Doc and a Western blot analysis was performed. Our results revealed a significant overexpression of HO-1 levels following Doc treatment, indicating that RM-1 cells also upregulate HO-1 in response to chemotherapy (*P* < 0.05) (Fig. 1A). This finding suggests a consistent mechanism across different PC cell lines, further supporting the rationale for targeting HO-1 in combination with Doc to enhance therapeutic efficacy.

### HO-1 inhibition and knockout decrease RM-1 cell viability and enhance chemotherapy sensitivity *in vitro*

We next investigated the effect of HO-1 inhibition on RM-1 cell viability and response to Doc. To evaluate the synergistic effect of combining HO-1 inhibition using ZnPP with Doc, we treated RM-1 cells *in vitro* with ZnPP, Doc, and their combination. Our results demonstrated that the combined treatment significantly decreased cellular viability and increased RM-1 cell sensitivity to Doc across various Doc concentrations and at different seeding densities (*P* < 0.05) (Fig. 1B and C). Similarly, SnPP combined with Doc at different concentrations enhanced RM-1 chemosensitivity and reduced cell survival compared to each treatment alone and to the control (*P* < 0.05) (Fig. 1d).

To further confirm the role of HO-1 inhibition on cell viability, we generated an HO-1 KO RM-1 cell line using lentiviral transfection. The KO phenotype was confirmed by western blot analysis, with CoPP serving as a positive control for HO-1 induction (*P* < 0.05) (Fig. 1E). The transfection specifically knocked out HO-1 protein without affecting its constitutive form, HO-2, as confirmed by western blot analysis (*P* < 0.05) (Fig. 1E). Our results indicated that HO-1 KO significantly reduced cell viability compared to parental cells at the same seeding density (*P* < 0.05) (Fig. 1F). Additionally, HO-1 KO markedly enhanced the chemosensitivity of RM-1 cells, both when normalized to their own control and to the parental control (*P* < 0.05) (Fig. 1G). Together, these data confirm our previous findings that HO-1 inhibition increases PC cell sensitivity to chemotherapy and reduces cell viability *in vitro*.

### HO-1 inhibition enhances PC cell chemosensitivity *in vivo*

**Fig. 2:**
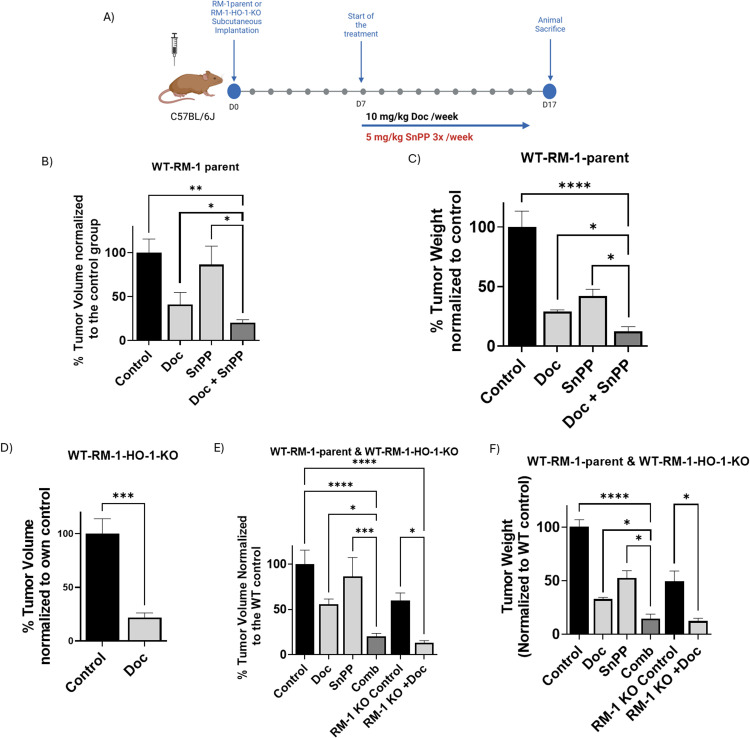
*In vivo* assessment of targeting HO-1 using a subcutaneous mouse model of PC. (A) Six-week-old male WT C57Bl/6 J mice were subcutaneously implanted with either RM-1 parental or RM-1-HO-1-KO cells. On day 7, post tumor formation, mice were randomized into treatment groups and received intraperitoneal injections. WT-RM-1-parent mice were allocated into 4 groups: group 1 (vehicle control), group 2 (10 mg/kg Doc once weekly), group 3 (5 mg/kg SnPP three times weekly), and group 4 (both Doc and SnPP). WT-RM-1-HO-1-KO mice were divided into 2 groups: control and Doc treatment. (B) A significant reduction in tumor volume was observed in the combination treatment compared to individual treatments and control. (C) Tumor weight significantly decreased with the combination treatment compared to individual treatments and control. (D) WT-RM-1-HO-1-KO groups demonstrated increased sensitivity to Doc, indicated by reduced % tumor volume compared to their own control. (E) Significant reduction in tumor volume in WT-RM-1-HO-1-KO groups normalized to WT-RM-1-parent control group. (F) Significant reduction in tumor weight in the WT-RM-1-HO-1 KO group treated with Doc normalized to WT-RM-1-parent control group. (*n* = 5, **** = P<0.0001, *** = P<0.0001, ** = P<0.001, * = P<0.05).

Given the decrease in cell viability *in vitro* with HO-1 inhibition, we next evaluated the impact of combining HO-1 inhibition with Doc on PC tumor growth *in vivo*. RM-1 cells were subcutaneously implanted into the right flank of wild-type C57BL/6 J mice, and tumor growth was monitored throughout the entire period of the experiment (*P* < 0.05) (Fig. 2A). Our results showed that HO-1 inhibition significantly suppressed tumor growth, leading to a marked reduction in tumor volume and tumor weight in the combined treatment group compared to each treatment alone and the control group (*P* < 0.05) (Fig. 2B and C).

To further confirm the effect of HO-1 inhibition on PC response to Doc *in vivo*. RM-1 KO cells were also subcutaneously implanted into the right flank of wild-type C57BL/6 J mice and tumor growth was monitored over the study period (*P* < 0.05) (Fig. 2A). HO-1 KO RM-1 significantly reduced tumor cell growth and augmented PC tumor sensitivity to Doc. The group treated with Doc showed a significant reduction in tumor volume and weight compared to the control group (*P* < 0.05) (Fig. 2D). Normalizing the results to the WT control group further demonstrated the remarkable tumor suppression effect of HO-1 KO on tumor growth in the control group implanted with HO-1 KO RM-1 and in the group treated with Doc compared to the WT groups (*P* < 0.05) (Fig. 2E and F).

Together, these results strongly suggest the efficacy of HO-1 inhibition in reducing PC tumor cells proliferation and increasing sensitivity to chemotherapy.

### HO-1 inhibition combined with Doc reduces Ki67 expression and increases CC3 expression in tumor tissues

**Fig. 3:**
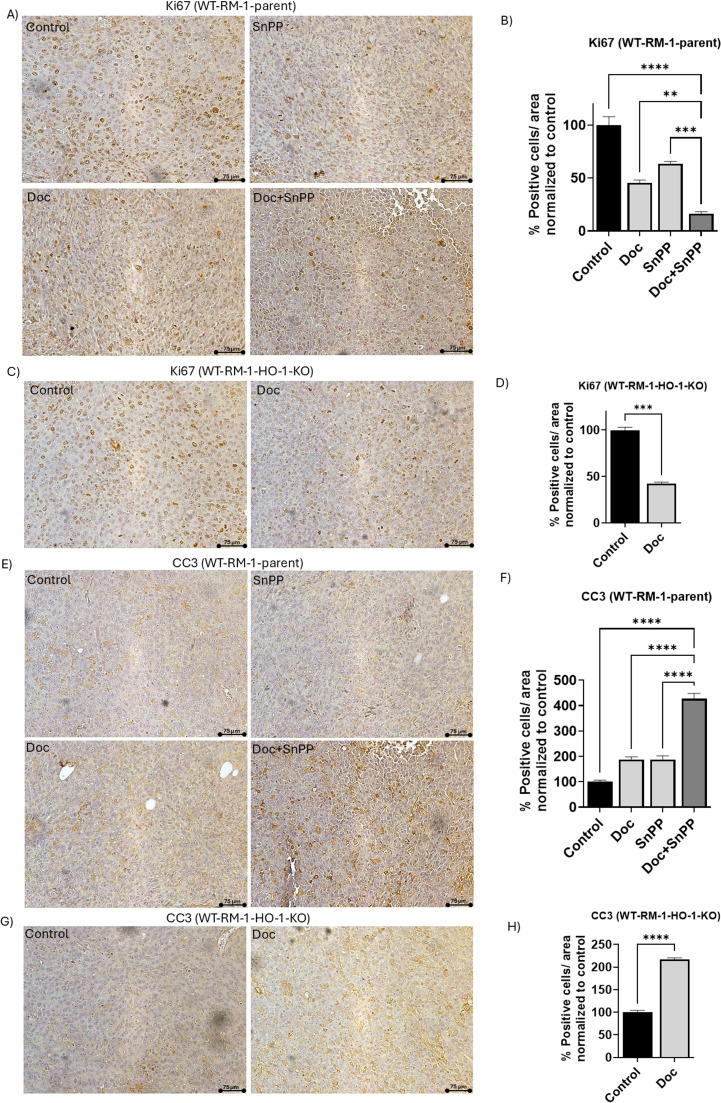
Combination treatment of HO-1 inhibitors with Doc reduces Ki67 expression and increases CC3 expression in PC tissues. (A) Representative images of Ki67 protein IHC staining in WT-RM-1-parent PC tumor tissues from the four groups: control, Doc, SnPP, Doc+SnPP. (B) Quantitative analysis of Ki67 protein expression, presented as the percentage of positively stained cells normalized to the control, demonstrating a decrease in Ki67 expression in the combination treatment group compared to individual treatment and control in WT-RM-1-parent group. (C) Representative images of Ki67 protein IHC staining in WT-RM-1-HO-1-KO tumor tissues from the control and Doc-treated groups. (D) Quantitative analysis of Ki67 protein expression in WT-RM-1-HO-1-KO tissues, demonstrating a significant decrease in percentage of positively stained cells in the Doc-treated group compared to control. (E) Representative images of CC3 protein IHC staining in PC tumor tissues collected from WT-RM-1-parent groups. (F) Quantitative analysis of CC3 protein expression, showing a significant increase in the percentage of positively stained cells in the combination treatment group compared to control in WT-RM-1-parent groups. (G) Representative images of CC3 IHC staining in PC tumor tissues from WT-RM-1-HO-1-KO groups. (H) Quantitative analysis of CC3 protein expression, showing significant increase in the percentage of positively stained cells in the Doc-treated group compared to control in WT-RM-1-HO-1-KO groups. (*n* = 5, **** = P<0.0001, *** = P<0.0001, ** = P<0.001).

Since our *in vivo* studies revealed a significant reduction in tumor growth in the combined treatment group of Doc and SnPP compared to the other groups, we investigated the effect of the HO-1 inhibition on the expression level of Ki67, a biomarker strongly associated with cell proliferation and progression in PC [65]. IHC analysis of the tumor tissues of the WT-RM-1-parent groups revealed that the combined treatment group exhibited significantly lower Ki67 expression levels compared to the control group and the groups that received individual treatment (*P* < 0.05) (Fig. 3A and B).

Similarly, IHC analysis of tumor tissues from the WT-RM-1-HO-1-KO group showed that the group treated with Doc had significantly lower Ki67 expression levels compared to the control group (*P* < 0.05) (Fig. 3C and D). This confirms our hypothesis that HO-1 inhibition, in combination with Doc reduces PC cell proliferation and enhances the response to Doc treatment.

We previously demonstrated that HO-1 inhibition sensitizes PC cells to Doc-induced apoptosis *in vitro* [64]. To further investigate this, we examined the effect of HO-1 inhibition on CC3 expression, a biomarker for apoptosis, in tumor tissues using IHC analysis. Our results indicated that HO-1 inhibition significantly increased CC3 expression levels in the WT-RM-1-parent combined treatment group compared to the control group and the groups that received individual treatments (*P* < 0.05) (Fig. 3E and F). Additionally, the WT-RM-1-HO-1-KO group treated with Doc exhibited significantly higher CC3 expression levels compared to its control group (*P* < 0.05) (Fig. 3G and H).

These collective findings suggest that HO-1 inhibition reduces tumor cell proliferation through the downregulation of Ki67 and increase PC sensitivity to Doc, as evidenced by the increased CC3 expression.

### HO-1 inhibition increases CD4^+^ and CD8^+^ T cells infiltration in tumor tissues

**Fig. 4:**
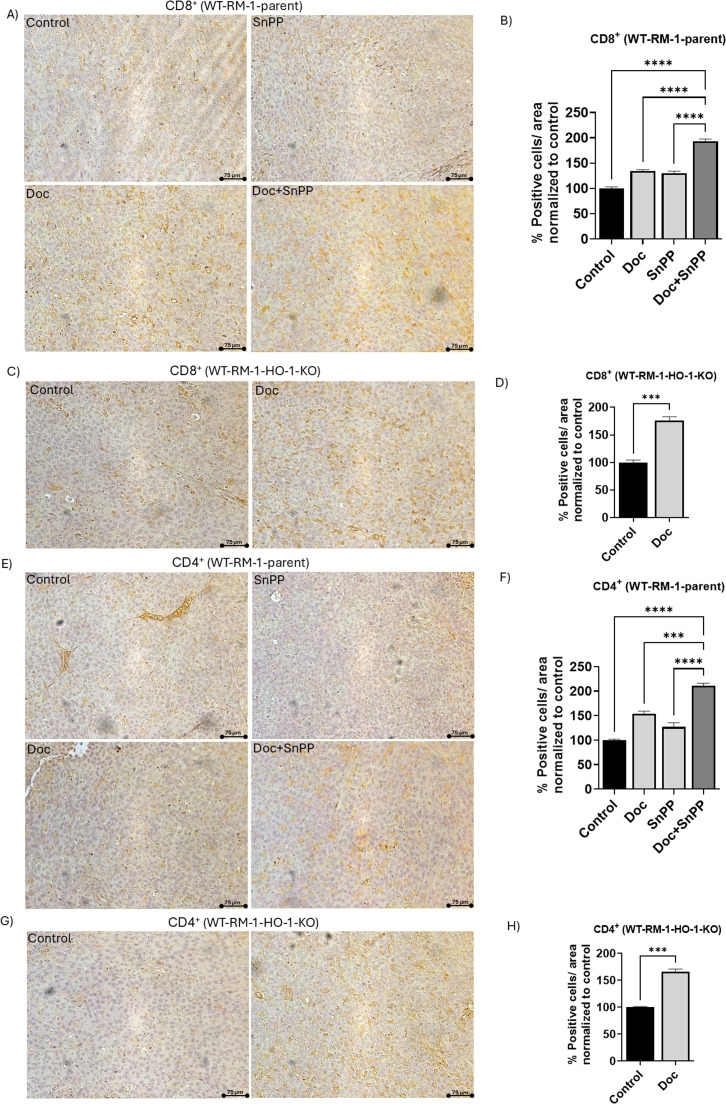
HO-1 inhibition increases CD4^+^ and CD8^+^ T cells infiltration in PC tumor tissues. (A) Representative images of CD8^+^ IHC staining in WT-RM-1-parent PC tumor tissues from the four groups: control, Doc, SnPP, Doc+SnPP. (B) Quantitative analysis of CD8^+^T cells, presented as the percentage of positively stained cells normalized to the control, showing an increase in CD8^+^ T cells infiltration in the combination treatment group compared to individual treatment and control in WT-RM-1-parent group. (C) Representative images of CD8^+^ IHC staining in WT-RM-1-HO-1-KO tumor tissues from the control and Doc treatment groups. (D) Quantitative analysis of CD8^+^ T cells in WT-RM-1-HO-1-KO tissues, demonstrating a significant increase in the percentage of positively stained cells in the Doc-treated group compared to control. (E) Representative images of CD4^+^ IHC staining in PC tumor tissues from WT-RM-1-parent groups. (F) Quantitative analysis of CD4^+^ T cells showing a significant increase in the percentage of positively stained cells in the combination treatment group compared to control in WT-RM-1-parent groups. (G) Representative images of CD4^+^ IHC staining in PC tumor tissues from WT-RM-1-HO-1-KO groups. (H) Quantitative analysis of CD4^+^ T cells, showing a significant increase in the percentage of positively stained cells in the Doc-treated group compared to control in WT-RM-1-HO-1-KO groups. (*n* = 5, **** = P<0.0001, *** = P<0.0001).

Evaluating T cell infiltration in tumor tissues is crucial for assessing therapy effectiveness, as T cells play a key role in the immune response to cancer. High levels of T cell infiltration are associated with a better prognosis and improved therapy response. We began by examining CD8^+^ T cells infiltration in the tumor tissues, as these are the most important effector T cells in the tumor microenvironment. Our data showed that HO-1 inhibition significantly increased CD8^+^ T cells infiltration in the tumor tissues of the combined treatment group in WT-RM-1-parent mice, compared to each treatment alone and to the control group (*P* < 0.05) (Fig. 4A and B). Similarly, tissues from WT-RM-1-HO-1-KO mice exhibited a significant increase in CD8^+^ T cells infiltration in the group treated with Doc compared to the control group (*P* < 0.05) (Fig. 4C and D).

Then, we focused on evaluating CD4^+^ T cells infiltration, given their important role in orchestrating the immune response within the tumor microenvironment. Our results showed that HO-1 inhibition significantly increased CD4^+^ T cell infiltration in the tumor tissues of the combined treatment group in WT-RM-1-parent mice, compared to each treatment alone and the control group (*P* < 0.05) (Fig. 4E and F). Additionally, tumor tissues from WT-RM-1-HO-1-KO mice showed a similar enhancement in CD4^+^ T cells infiltration in the Doc-treated group compared to the control group (*P* < 0.05) (Fig. 4G and H).

These collective observations strongly suggest that HO-1 inhibition increases CD4^+^ and CD8^+^ T cells infiltration in the tumor tissues. This increased infiltration indicates a more robust immune response against cancer cells, potentially contributing to better therapeutic outcomes and improved prognosis.

### HO-1 inhibition shifts macrophage polarization toward an M1 phenotype *in vitro*

**Fig. 5:**
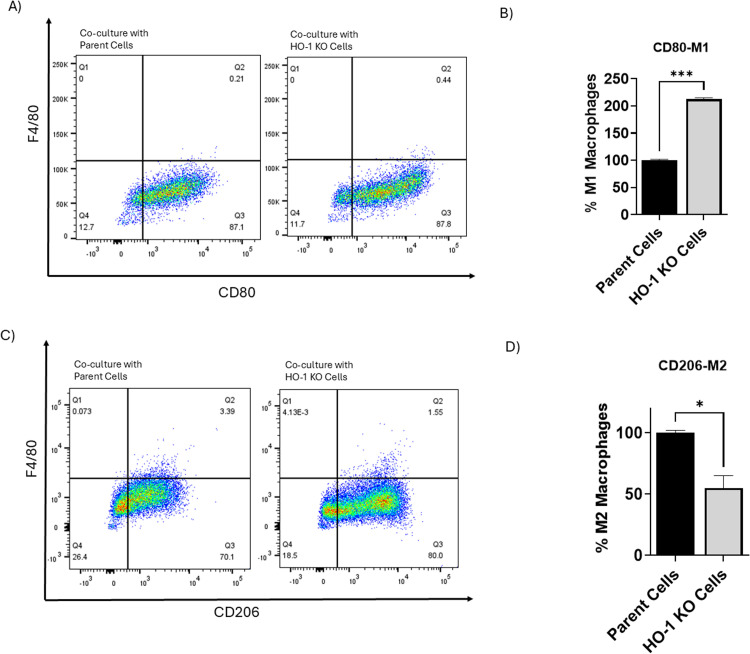
HO-1 inhibition promotes M1 macrophage polarization *in vitro*. (A) Flow cytometry analysis of M1 macrophages, identified by F4/80+ and CD80+ markers, showing a significant increase in M1 phenotype in U937 cells co-cultured with DU145 HO-1 KO cells compared to those co-cultured with DU145 parent cells. (B) Quantitative analysis of F4/80+ and CD80+ cells in the two groups. (C) Flow cytometry analysis of M2 macrophages, identified by F4/80+ and CD206+ markers, demonstrating a significant decrease in M2 phenotype in U937 cells co-cultured with DU145 HO-1 KO cells compared to those co-cultured with DU145 parent cells. (D) Quantitative analysis of F4/80+ and CD206+ cells in the two groups. (*n* = 3, *** = P<0.0001, * = P<0.05).

In PC, TAMs are the most abundant population of inflammatory cells infiltrating PC tissues, and they play a key role in tumor progression and resistance to therapies [66,67]. Next, we focused our attention on evaluating the effect of HO-1 inhibition on the macrophage polarization. *In vitro*, we used conditioned media from WT parent cells and HO-1 KO cells to assess the effect of cytokines produced by these cells on the polarization of U937 monocytes. Flow cytometry analysis indicated that the U937 cells cultured in contact with the HO-1 KO conditioned media had a significantly higher levels of M1 phenotype, represented by F4/80+ and CD80+ cells (*P* < 0.05) (Fig. 5A and B), and a lower levels of M2 phenotype, represented by F4/80+ and CD206+ cells (*P* < 0.05) (Fig. 5C and D), compared to cells cultured in contact with the WT parent conditioned media. These findings suggest that HO-1 inhibition shifts TAMs polarization toward an M1 phenotype *in vitro*, which may enhance the anti-tumor immune response.

### HO-1 inhibition combined with Doc promotes M1 phenotype while maintaining M2 levels *in vivo*

**Fig. 6:**
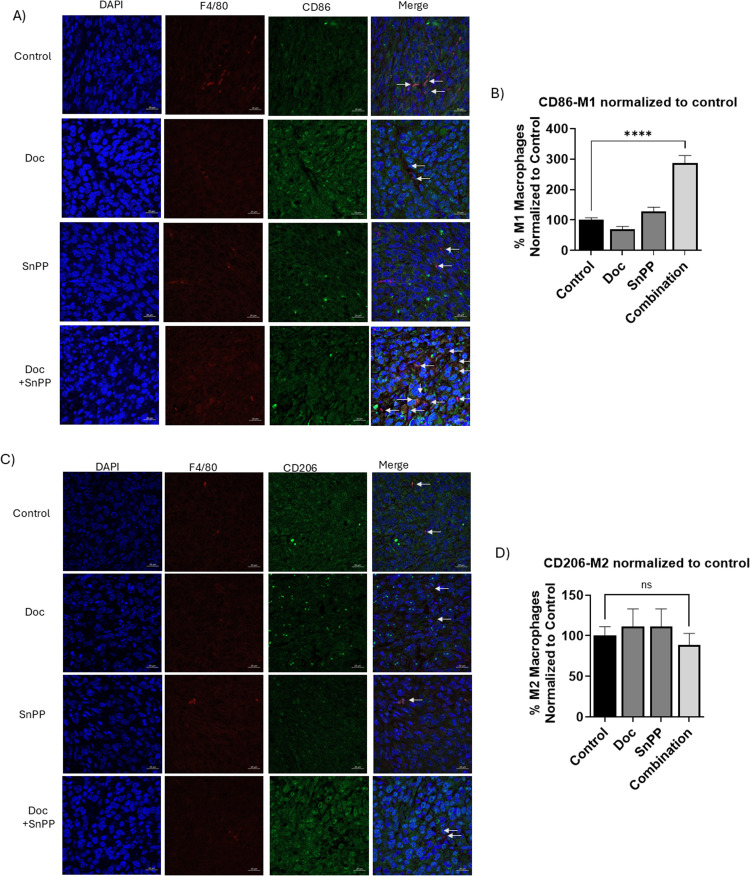
Combined treatment with HO-1 inhibitors and Doc enhances M1 macrophage polarization without affecting M2 levels in PC tissues. (A) Representative images of M1 macrophages, indicated by F4/80+ and CD86+ IF staining in WT-RM-1-parent PC tumor tissues from the four groups: control, Doc, SnPP, Doc+SnPP. (B) Quantitative analysis of M1 macrophages, presented as the percentage of positively co-stained cells normalized to the control. Results show a significant increase in M1 phenotype polarization in the combination treatment group compared to individual treatments and control in WT-RM-1-parent group. (C) Representative images of M2 macrophages, indicated by F4/80+ and CD206+ IF staining in WT-RM-1-parent tumor tissues. (D) Quantitative analysis of M2 macrophages in WT-RM-1-parent tissues, demonstrating a consistent level of M2 macrophages across the four groups. (*n* = 5, **** = *P<0.0001).*

Our *in vitro* results prompted us to investigate the shift in TAMs polarization within the tumor tissues from the different treatment groups of WT-RM-1-parent mice. IF analysis of these tumor tissues indicated that HO-1 inhibition combined with Doc significantly increased the number of M1 phenotype macrophages, represented by F4/80+ and CD86+ cells, compared to each treatment alone and the control group (*P* < 0.05) (Fig. 6A and B). However, the levels of M2 macrophages, represented by F4/80+ and CD206+ cells, remained unchanged across all four groups (*P* < 0.05) (Fig. 6C and D). These findings suggest that combining HO-1 inhibition with Doc induces an anti-tumor immune response in PC, potentially leading to better therapeutic outcomes.

### Macrophage-specific HO-1 inhibition enhances anti-tumor effects and chemotherapy sensitivity and enhances M1 polarization

**Fig. 7:**
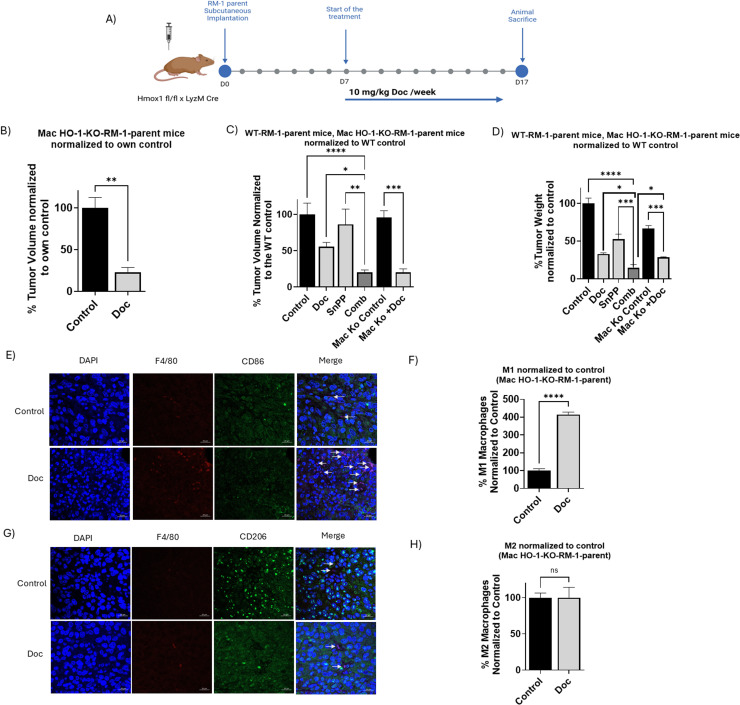
*In vivo* macrophage specific HO-1 inhibition enhances PC tumor response to chemotherapy and enhance M1 polarization. (A) Six-week-old male Hmox1 fl/fl x LyzM Cre (Mac HO-1 KO) mice were subcutaneously implanted with RM-1 parent cells. When tumor formed, around day7, mice were randomly assigned into treatment groups and received intraperitoneal injections. Mac HO-1-KO-1-RM-1-parent mice were allocated into two groups: group 1 (vehicle control) and group 2 (10 mg/kg Doc once weekly). (B) A significant reduction in tumor volume was observed in Mac HO-1-KO-1-RM-1-parent in the Doc-treated group normalized to own control. (C) Significant reduction in tumor volume in the Doc-treated group of Mac HO-1-KO-1-RM-1-parent mice when normalized to WT-RM-1-parent control. (D) A significant reduction in tumor weight in the Doc-treated group of Mac HO-1-KO-1-RM-1-parent mice when normalized to WT-RM-1-parent control. (E) Representative images of M1 macrophages, indicated by F4/80+ and CD86+ IF staining in Mac HO-1-KO-1-RM-1-parent PC tumor tissues. (F) Quantitative analysis of M1 macrophages, presented as the percentage of positively co-stained cells normalized to the control, showing a significant increase in M1 phenotype polarization in the Doc-treated group compared to control in Mac HO-1-KO-RM-1-parent group. (C) Representative images of M2 macrophages, indicated by F4/80+ and CD206+ IF staining in Mac HO-1-KO-RM-1-parent tumor tissues. (D) Quantitative analysis of M2 macrophages in Mac HO-1-KO-RM-1-parent tissues, demonstrating a consistent level of M2 macrophages in both groups. (*n* = 5, **** = P<0.0001, ** = P<0.001, * = P<0.05).

Next, we sought to determine whether the shift in TAMs polarization toward an M1 phenotype is a result of HO-1 inhibition is solely responsible for the observed anti-tumor effects and increased sensitivity to Doc, as compared to the results obtained with systemic HO-1 inhibition using SnPP. For this purpose, we conducted an *in vivo* experiment using Hmox1 fl/fl x LyzM Cre mice, which have an HO-1 KO specifically in their macrophages. These mice were kindly provided by Dr. Barbara Wegiel from Harvard Medical School. We included a control group and a group treated with Doc (Fig. 7A).

Our results demonstrated that the Doc-treated group exhibited a significantly smaller tumor volume compared to its corresponding control group (*P* < 0.05) (Fig. 7B). When these results were normalized to the WT-RM-1-parent group, the substantial impact of HO-1 knockout macrophages on reducing tumor volume and weight became even more apparent (*P* < 0.05) (Fig. 7C and D). Although the response was significant, it was not as pronounced as in the WT-RM-1-parent combined treatment group.

We also evaluated the levels of M1 *versus* M2 macrophages using IF analysis and found similar results to our WT-RM-1 group analysis. The Mac HO-1-KO mice treated with Doc exhibited significantly higher levels of the M1 phenotype, represented by F4/80+ and CD86+ cells, compared to the control (*P* < 0.05) (Fig. 7E and F), while M2 levels, represented by F4/80+ and CD206+ cells, remained unchanged (Fig. 7G and H).

In conclusion, these findings suggest that macrophage-specific HO-1 inhibition significantly contributes to M1 shift and to the anti-tumor immune response and enhances sensitivity to Doc, though systemic HO-1 inhibition provides a more robust therapeutic outcome.

## Discussion

The high fatality rate in advanced PC is attributed to therapy resistance and poor prognosis [68]. HO-1 contributes to PC tumor survival and progression through various mechanisms. The byproducts of heme degradation, particularly CO and free iron, have been shown to possess anti-apoptotic, pro-angiogenic, and pro-metastatic properties [69,70]. Furthermore, HO-1 facilitates immune evasion and modulates the tumor microenvironment to support cancer cell proliferation and resistance to apoptosis [31,62]. Elevated HO-1 levels have been linked to PC progression, with significant upregulation observed in hormone refractory PC (HRPC) tissues compared to benign or localized PC [71]. These characteristics make HO-1 a compelling target for therapeutic intervention.

Our lab previously demonstrated that HO-1 inhibition reduces PC cell survival and sensitizes PC cells to Doc *in vitro* through various interconnected mechanisms [64]. In this study, we investigated the potential of HO-1 inhibition on PC cell survival and sensitivity to Doc *in vivo* to further validate these effects. We also assessed the impact of HO-1 inhibition on immune infiltration to better understand the therapy response of the combined treatment in PC.

Our results showed that the induction of HO-1 by chemotherapy in RM-1 cells aligns with previous findings in human PC cells, indicating a conserved response across different PC models. This overexpression likely represents a cellular defense mechanism against the oxidative stress induced by chemotherapy. However, this protective response can inadvertently contribute to chemoresistance, highlighting the need for strategies that can modulate HO-1 activity to enhance the efficacy of chemotherapeutic agents.

Our *in vitro* experiments revealed that both ZnPP and SnPP, as well as HO-1 KO, significantly decreased cell viability and enhanced the sensitivity of RM-1 cells to Doc. This synergistic effect was further corroborated *in vivo*, where the combination of SnPP and Doc led to a marked reduction in tumor growth compared to either treatment alone. These results emphasize the potential of HO-1 inhibition to overcome chemoresistance and improve therapeutic responses in PC.

The significant reduction in Ki67 expression, a marker closely linked to cell proliferation, alongside the increase in CC3 levels, an indicator of apoptosis, in tumor tissues from the combined treatment group suggests that HO-1 inhibition not only suppresses tumor cell proliferation but also promotes apoptosis. This dual action highlights the complex role of HO-1 in tumor progression and reinforces its potential as a therapeutic target for disrupting both proliferation and survival pathways in cancer cells.

One of the most significant findings of our study is the enhanced infiltration of CD4^+^ and CD8^+^ T cells in tumor tissues following HO-1 inhibition. This suggests that targeting HO-1 can modulate the immune microenvironment to support a more robust anti-tumor immune response. The increased presence of cytotoxic T cells is particularly promising, as these are critical for direct tumor cell killing and are often associated with better clinical outcomes [42]. The increased T cells infiltration following HO-1 inhibition not only supports direct anti-tumor cytotoxicity but also promotes a more robust and sustained immune response against the cancer. This immune modulation, combined with the direct cytotoxic effects of HO-1 inhibitors, can lead to a synergistic enhancement of therapeutic efficacy when used in conjunction with chemotherapy.

Moreover, our results indicate that HO-1 plays a crucial role in macrophage polarization within the tumor microenvironment. The shift towards an M1 phenotype, observed both *in vitro* and *in vivo*, suggests that HO-1 inhibition can reprogram TAMs to support anti-tumor immunity and potentially improve therapeutic outcomes. This reprogramming could counteract the pro-tumorigenic functions of M2 macrophages, thereby disrupting tumor-promoting processes such as angiogenesis, tissue remodeling, and immunosuppression. By increasing M1 polarization in the combination treatment group, HO-1 inhibitors can alter the tumor microenvironment to favor immune-mediated tumor eradication, further supporting the rationale for their use in combination with chemotherapy.

While both systemic and macrophage-specific HO-1 inhibition enhanced the anti-tumor effects of Doc, systemic inhibition at an optimal dosage is likely to achieve a more robust therapeutic outcome. This enhanced efficacy may be attributed to the broader influence of systemic inhibition on various cell types within the tumor microenvironment, including not only cancer cells but also other key immune cells, thereby creating a more comprehensive disruption of tumor growth and survival pathways.

Our findings highlight the therapeutic potential of HO-1 inhibition in PC, particularly in combination with Doc. By reducing tumor proliferation, enhancing apoptosis, and modulating the tumor immune microenvironment, HO-1 inhibitors can significantly improve treatment responses and overcome chemoresistance. These results pave the way for further preclinical investigation of HO-1 inhibitors as part of combination therapies for advanced PC.

Despite significant advances in understanding HO-1′s role in PC, several critical knowledge gaps remain. First, the precise molecular pathways by which HO-1 modulates therapy resistance and immune evasion are not fully elucidated. While the enzymatic byproducts of HO-1 activity, such as CO and biliverdin, are known to influence tumor growth and angiogenesis, the non-canonical roles of HO-1, including its nuclear localization and effects on gene expression, require further investigation. Additionally, the interplay between HO-1 and other tumor-promoting factors within the microenvironment, such as cytokines, immune checkpoints, and metabolic pathways, remains underexplored.

Another challenge lies in determining the optimal conditions for HO-1 inhibition in combination therapies. Questions about dosage, timing, and potential off-target effects need to be addressed to maximize therapeutic efficacy while minimizing toxicity. Furthermore, the variability in HO-1 expression across patient populations and tumor subtypes adds complexity to its clinical application, necessitating the identification of predictive biomarkers to identify patients who would benefit most from HO-1-targeted interventions.

To bridge these gaps, future studies should leverage advanced multi-omics approaches, including transcriptomics, metabolomics, and proteomics, to map the downstream targets and pathways influenced by HO-1. Employing patient-derived xenografts and genetically engineered mouse models can better capture the heterogeneity of human tumors and enable the evaluation of combination therapies under clinically relevant conditions.

The development of more selective and bioavailable HO-1 inhibitors, coupled with innovative delivery systems such as nanoparticle-based carriers, will enhance the clinical applicability of these agents over the next few years. Combining HO-1 inhibitors with emerging immunotherapies, including immune checkpoint inhibitors and CAR-T cell therapies, holds promise for amplifying anti-tumor immune responses and overcome resistance.

Lastly, as the role of the tumor microenvironment in therapy resistance becomes clearer, targeting HO-1′s influence on macrophage polarization and immune suppression will likely emerge as a cornerstone of combination therapies. By modulating the tumor immune landscape, these strategies could transform HO-1 inhibition from a supportive therapy to a central component of PC treatment regimens.

The future of HO-1′s research lies in its integration with precision oncology and immunotherapy, offering a pathway to overcome therapeutic resistance and improving outcomes for patients with advanced PC. As these efforts progress, multidisciplinary collaboration will be essential to translate these findings into effective clinical therapies that address the urgent unmet needs in PC management.

## CRediT authorship contribution statement

**Ramia J. Salloom:** Writing – review & editing, Writing – original draft, Visualization, Validation, Supervision, Project administration, Methodology, Investigation, Formal analysis, Data curation, Conceptualization. **Dania Z. Sahtout:** Data curation. **Iman M. Ahmad:** Writing – review & editing. **Maher Y. Abdalla:** Writing – review & editing, Validation, Supervision, Project administration, Conceptualization.

## Declaration of competing interest

The authors declare that they have no known competing financial interests or personal relationships that could have appeared to influence the work reported in this paper.

## Appendix Supplementary materials

Image, application 1
