# Supplementary material for: Synergistic effects of HO-1 inhibition and chemotherapy on tumor proliferation and immune infiltration: An in vitro and in vivo approach to enhancing prostate cancer treatment
Source: Transl Oncol. 2025 Mar 3;54:102339. doi: 10.1016/j.tranon.2025.102339 (PMC11925535; doi:10.1016/j.tranon.2025.102339)
Supplement: Supplementary file 1 [file mmc1.pptx]

## Slide 1
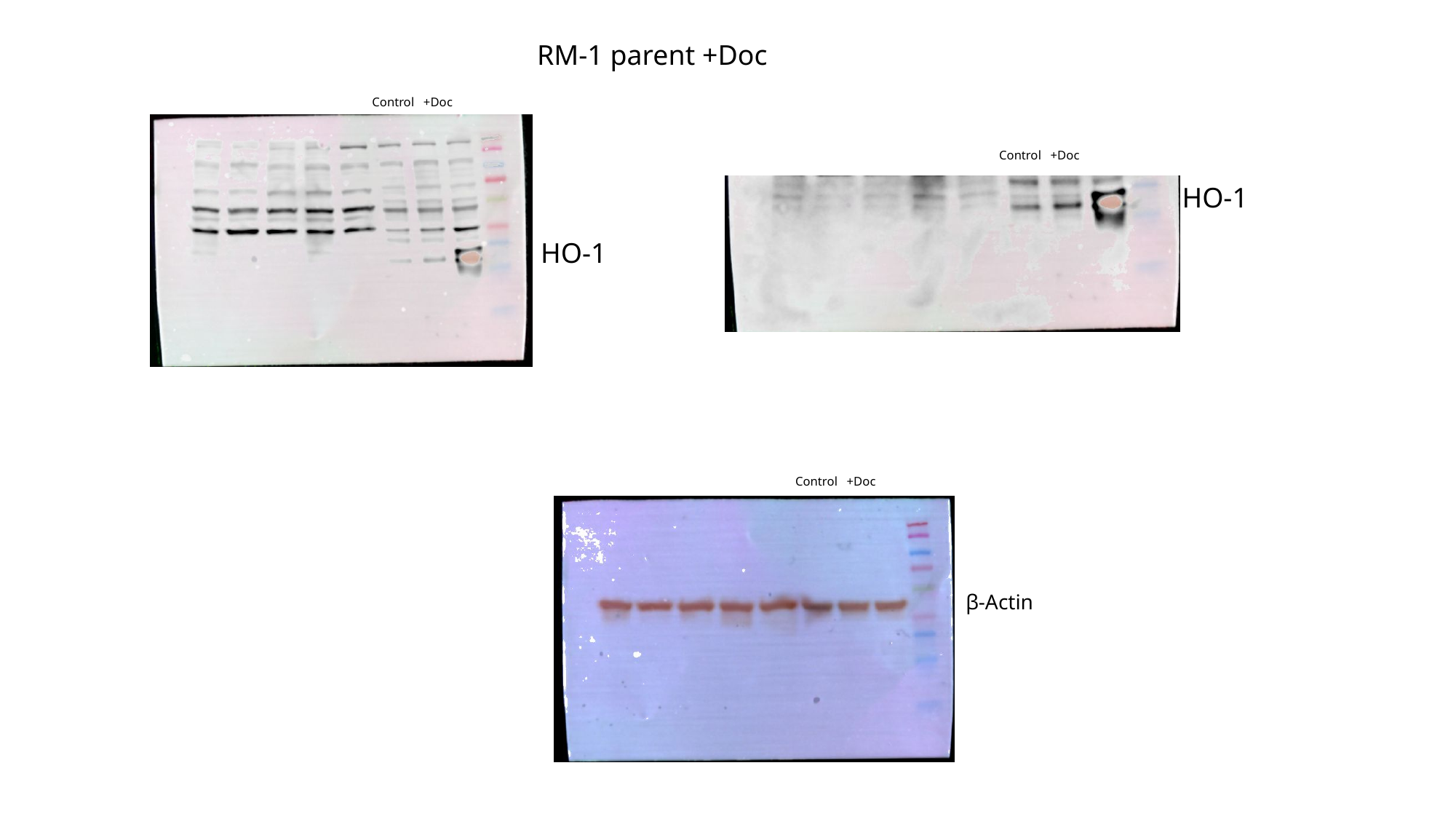

RM-1 parent +Doc
Control +Doc
Control +Doc
HO-1
HO-1
Control +Doc
β-Actin

## Slide 2
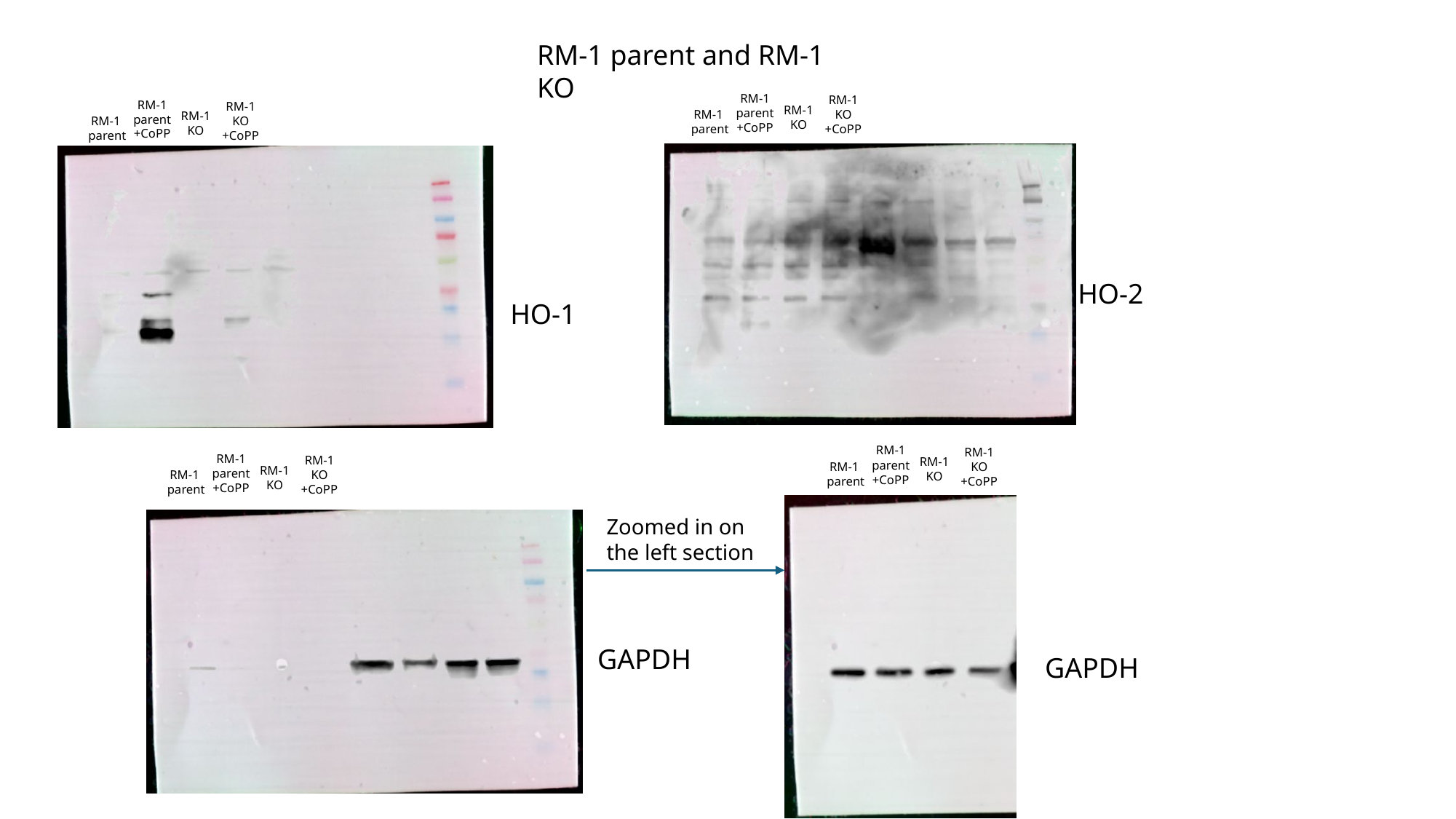

RM-1 parent and RM-1 KO
RM-1 parent
+CoPP
RM-1 KO +CoPP
RM-1 parent
+CoPP
RM-1 KO +CoPP
RM-1 KO
RM-1
parent
RM-1 KO
RM-1
parent
HO-2
HO-1
RM-1 parent
+CoPP
RM-1 KO +CoPP
RM-1 parent
+CoPP
RM-1 KO +CoPP
RM-1 KO
RM-1
parent
RM-1 KO
RM-1
parent
Zoomed in on the left section
GAPDH
GAPDH
